# Should Metabolic Diseases Be Systematically Screened in Nonsyndromic Autism Spectrum Disorders?

**DOI:** 10.1371/journal.pone.0021932

**Published:** 2011-07-07

**Authors:** Manuel Schiff, Jean-François Benoist, Sofiane Aïssaoui, Odile Boepsflug-Tanguy, Marie-Christine Mouren, Hélène Ogier de Baulny, Richard Delorme

**Affiliations:** 1 APHP, Reference Center for Inherited Metabolic Disease, Hôpital Robert Debré, Paris, France; 2 APHP, Child Neurology and Metabolic Disease department, Hôpital Robert Debré, Paris, France; 3 Inserm U676, Paris, France; 4 Institut Pasteur, Human Genetics and Cognitive Functions, Paris, France; 5 APHP, Child Psychiatry department, Hôpital Robert Debré, Paris, France; 6 Université Paris 7, Faculté de médecine Denis Diderot, IFR02, Paris, France; The University of Western Australia, Australia

## Abstract

**Background:**

In the investigation of autism spectrum disorders (ASD), a genetic cause is found in approximately 10–20%. Among these cases, the prevalence of the rare inherited metabolic disorders (IMD) is unknown and poorly evaluated. An IMD responsible for ASD is usually identified by the associated clinical phenotype such as dysmorphic features, ataxia, microcephaly, epilepsy, and severe intellectual disability (ID). In rare cases, however, ASD may be considered as nonsyndromic at the onset of a related IMD.

**Objectives:**

To evaluate the utility of routine metabolic investigations in nonsyndromic ASD.

**Patients and Methods:**

We retrospectively analyzed the results of a metabolic workup (urinary mucopolysaccharides, urinary purines and pyrimidines, urinary creatine and guanidinoacetate, urinary organic acids, plasma and urinary amino acids) routinely performed in 274 nonsyndromic ASD children.

**Results:**

The metabolic parameters were in the normal range for all but 2 patients: one with unspecific creatine urinary excretion and the other with persistent 3-methylglutaconic aciduria.

**Conclusions:**

These data provide the largest ever reported cohort of ASD patients for whom a systematic metabolic workup has been performed; they suggest that such a routine metabolic screening does not contribute to the causative diagnosis of nonsyndromic ASD. They also emphasize that the prevalence of screened IMD in nonsyndromic ASD is probably not higher than in the general population (<0.5%). A careful clinical evaluation is probably more reasonable and of better medical practice than a costly systematic workup.

## Introduction

Autism spectrum disorders (ASD) are neurodevelopmental disabilities characterized by an onset before three years of age, a severe and pervasive impairment in reciprocal socialization, qualitative impairment in communication, restricted interests and repetitive behaviors [1]. The pathophysiology of ASD is complex, highly genetic and multifactorial [2]. Currently, genetic causes (chromosomal abnormalities or genetic alterations) of ASD can be identified in about 10–20% of cases [3]. Among these, the contribution of the rare inherited metabolic disorders (IMD) remains poorly evaluated. ASD are frequently reported in IMD with neuro-psychiatric involvement. In these situations, the IMD responsible for ASD is usually identified by associated clinical signs such as dysmorphic features, ataxia, microcephaly, epilepsy, and intellectual disability (ID) [4], [5]. In rare situations, however, ASD may appear as an isolated disorder at the onset of a few IMDs (e.g. mainly untreated phenylketonuria, classical homocystinuria, Sanfilippo disease). The latter are important to identify as early as possible since some of them are amenable to treatment. In many child and adolescent psychiatry units, a metabolic screening is routinely performed in all ASD patients whatever the associated clinical phenotype. Specifically, in ASD patients considered as nonsyndromic, *i.e.* those with no additional clinical symptoms suggesting an IMD, the outcome of this practice has not been evaluated. The aim of the present study was thus to retrospectively explore the benefits of such a systematic screening in 274 ASD patients considered as nonsyndromic.

## Materials and Methods

### Study design and population

A retrospective cohort study was conducted, and all children included aged two to seventeen years, were hospitalized at the Robert Debré University Hospital (Paris, France), in two specialized units labelled as reference centers for autism by the French Health Ministry, and registered with a final diagnosis of ASD, between January 1, 2006 and December 31, 2010. To be enrolled in the study, patients were to be evaluated during three to seven days by a multidisciplinary team including child psychiatrists, child neurologists, geneticists, psychologists and speech therapists. Diagnosis of ASD was based on DSM-IV criteria (APA, 1994) and strong clinical consensus among experts at the end of the hospitalization. Patients already diagnosed with medical disorders, identifiable neurological syndromes or focal neurological signs were excluded (n = 66). All patients with abnormal genetic exams (high-resolution karyotyping, Fragile X syndrome, 15q11-q13, 22q11 and 22q13 deletions detected by fluorescence in situ hybridization) performed during the hospitalization were excluded (n = 9). Patients with insufficient available information to classify them as nonsyndromic were also excluded (n = 39). Associated ID was assessed with the Wechsler Intelligence Scale for Children - third or fourth edition or the Wechsler Preschool and Primary Scale of Intelligence. For patients with moderate to profound ID or those who were non verbal, the cognitive level was estimated with the Psycho-Educational-Profile Revised (PEP-R) or the Brunet-Lezine developmental test [6]. Depending on the results of these tests, patients were classified as high functioning ASD patients (intellectual quotient or developmental quotient above 70) or low functioning ASD patients.

### Metabolic parameters

The following tests were performed: urinary mucopolysaccharides (total glycosaminoglycan- DMB assay and electrophoresis), urinary purines and pyrimidines (liquid chromatography-diode array detector), urinary creatine and guanidinoacetate (tandem mass spectrometry), urinary organic acids (gas chromatography-mass spectrometry), plasma and urinary amino acids (liquid chromatography-ninhydrine), respectively screening for mucopolysaccharidoses, inborn errors of purine and pyrimidine metabolism, creatine deficiency syndromes, organic acidurias and aminoacidopathies.

### *SLC6A8* sequencing and creatine uptake assay

Mutation screening of *SLC6A8* and creatine uptake assays in cultured skin fibroblasts were performed as already reported [7], [8]. Creatine uptake was measured after incubation with 25 µM creatine. The measured intracellular creatine concentration was expressed in picomol creatine per microgram total protein. The incubations were performed in triplicate.

### Ethics

The ethics committee of Robert Debré University Hospital (APHP, 75019 Paris, France) reviewed and approved the study. For molecular exams, informed consent was obtained from probands (if possible) and parents. However, the parents of children did not provide explicit informed consent concerning the metabolic exams, because the French ethical legislation specifies that informed consent is automatically waived for retrospective studies using patient files. Thus, the ethics committee of Robert Debré University Hospital (APHP, 75019 Paris, France) specifically waived the need for consent concerning these metabolic exams.

## Results

Among the 274 ASD patients enrolled in the study (Table 1), only two patients showed persistent alterations in metabolic parameters (Table 2): one with a 3-methylglutaconic aciduria and the other with an elevated creatine urinary excretion (between 1.5 and 2 fold control values on 3 different measurements performed on a 2-year period). Both patients exhibited autism, the first with a moderate ID and the second one with a non verbal IQ in the normal range. For the second patient, guanidinoacetate level was normal both in plasma and urine as was plasma creatine level (data not shown). To check for a creatine transport defect, *SLC6A8* was sequenced from genomic DNA extracted from fibroblasts and creatine uptake was assayed in fibroblasts. These two analyses revealed no abnormalities, indicating no primary deficit in creatine transport.

**Table 1:** Demographic and clinical characteristics of the patients enrolled in the study.

	Patients	
	n = 274	%
Gender (male/female)	241/33	88/12
Mean age (± SD)	7.3±3.6	
ASD diagnosis		
*Autism*	182	66
*Asperger syndrome*	18	7
*PDD-nos*	74	27
Associated ID	139	51
*Mild to moderate ID*	92	34
*Severe to profound ID*	47	17

ID: intellectual disability; PDD-nos: Pervasive Developmental Disorder - Not Otherwise Specified.

**Table 2:** Metabolic tests performed in the study.

	Number of subjects screened for each test	Number (%) of subjects with abnormal test
Urinary mucopolysaccharides	236	0
Urinary purines and pyrimidines	238	0
Urinary creatine and guanidinoacetate	203	1 (0.5)
Urinary organic acids	247	1 (0.4)
Urinary amino acids	247	0
Plasma amino acids	267	0

## Discussion

Among the 274 patients with ASD considered as nonsyndromic, we were unable to detect any patients with mucopolysaccharidoses, abnormalities of purine and pyrimidine metabolism, creatine deficiency syndromes or aminoacidopathies. The only abnormalities found were a non-specific increase in urinary creatine excretion and a 3-methylglutaconic aciduria. These results suggest that the prevalence of screened IMD in association with ASD is low (<0.5%) and thus indicate the weak cost effectiveness of a systematic metabolic workup in ASD considered as nonsyndromic.

Very few IMDs may begin with isolated ASD as a prominent feature. For example, phenylketonuria (PKU) concerns the rare neonates born in developed countries who escaped from neonatal screening or the unscreened neonates born in countries for which PKU screening is not systematically performed. These can exhibit an isolated ASD over months or years that is usually secondarily associated with behavioral disorders, epilepsy and severe ID [9], [10]. Similarly, children with classical homocystinuria (HCY) due to cystathionine-β-synthase deficiency may exhibit isolated autism [11]. The clinical picture may later be enriched by the cardinal manifestations of HCY *i.e.* lens subluxation, vascular thrombosis and skeletal abnormalities [12]. Isolated ASD may also be observed at the onset of mucopolysaccharidosis type III (MPS III) or Sanfilippo disease [13], [14]. MPS III patients often present with severe behavioral disorders and subtle signs of slowly progressing cognitive impairment, primary including speech regression and loss of toilet training and further leading to a severe encephalopathy [15]. In addition, rare cases of urea cycle disorders, especially ornithine transcarbamylase deficiency have been reported in patients with nonsyndromic ASD [16]. In this case, ASD are usually part of recurrent bouts of neuro-psychiatric problems (behavioral disorders, ataxic gait) frequently associated with hepato-digestive abnormalities [17]. These rare IMDs for which ASD is an initial sign could justify a systematic minimal metabolic screening in ASD patients considered as nonsyndromic, especially because PKU, HCY and urea cycle disorders are treatable conditions for which early recognition and treatment are essential. Plasma amino acids (PKU, HCY, and urea cycle disorders), post prandial plasma ammonia level (urea cycle disorders) and urinary MPS profile (Sanfilippo disease) could be proposed. In these disorders, however, the clinical picture comprises with time additional signs such as severe behavioral disorders, cognitive regression, ocular, liver or other neurological signs. Careful reappraisal of the clinical signs is therefore warranted and crucial because sometimes the initial diagnosis of nonsyndromic ASD should be reconsidered in the presence of subtle symptoms. At the Robert Debré hospital, due to published yet controversial recommendations [18], [19], a systematic metabolic workup is performed in all patients with ASD, whatever the associated symptoms. These metabolic tests are not exhaustive and concern various diseases with syndromic and nonsyndromic ASD. Moreover, a complete screening for all of the IMD is not feasible in a standard hospital setting [20]. Targeted testing could more effectively identify IMD that require early treatment. Plasma and urine amino acid profiles allow the detection of PKU and HCY, while urinary organic acid profiles can identify all of the organic acidurias, some of which include autistic signs [21], [22]. In creatine deficiency syndromes, autistic features are mostly not isolated but associated with ID, speech delay and epilepsy [23], [24]. A few inborn errors of purine (especially adenylosuccinase deficiency) and pyrimidine (especially dihydropyrimidine dehydrogenase deficiency) metabolism [25] may include autism in association with psychomotor retardation, epilepsy and muscular wasting in adenylosuccinase deficiency [26].

The two abnormalities identified in our cohort are remarkable. The patient with persistent creatine urinary excretion may have had a mild and so far uncharacterized creatine transporter defect. Moreover, on brain magnetic resonance spectroscopy imaging, the patient had a low but discernible creatine peak whereas in creatine transport defects, the creatine peak is usually markedly decreased [27]. Normality of creatine uptake as well as *SLC6A8* investigations were not in favor of a primary disorder of creatine transport. Low creatine peak on brain spectroscopy has been reported in other neurogenetic disorders especially in abnormalities of white matter homeostasis [28]. Elevated creatine urinary excretion in the patient is most probably a false positive result that might be related to excessive creatine dietary intake (mainly due to high protein intake) as previously reported [29]. Unfortunately, we were unable to call the patient back for diet control. Three-methylglutaconic aciduria is a non-specific abnormality underlying a highly heterogeneous and poorly defined group of diseases named the 3-methylglutaconic acidurias [22]. Besides the 3-methylglutaconyl-CoA hydratase deficiency, an inborn error of leucine catabolism, the whole group of 3-methylglutagonic acidurias is most probably seen in the context of various mitochondrial dysfunctions [22]. Further analyses in this direction are underway in this patient.

Notably, a recent study pointed out that mitochondrial dysfunction may be involved in ASD [30]. In this report, plasma lactate determination was performed in a restricted sample of 69 patients with ASD. Twenty per cent of them displayed hyperlactatemia and 7% fulfilled the criteria for a disorder of oxidative phosphorylation (OXPHOS) [30]. This initial study has limitations as the autistic clinical phenotype was not well defined. More recently, a retrospective study of 25 patients with a primary diagnosis of nonsyndromic autism who were further determined to have enzyme- or mutation- defined OXPHOS deficiency showed that 96% of these patients actually exhibited clinical symptoms differentiating them from idiopathic autism [31]. These results suggest that careful clinical and biochemical reappraisal is warranted in patients with ASD initially considered as nonsyndromic, but also confirm that ASD patients with OXPHOS dysfunction often exhibit other symptoms such as microcephaly, marked motor delay, sensorineural deafness, oculomotor abnormalities, exercise intolerance, cardiomyopathy or renal tubular dysfunction. Accordingly, we decided not to screen for hyperlactatemia in our nonsyndromic ASD population. Furthermore, normal plasma lactic acid concentrations do not exclude the presence of a mitochondrial disorder [32], [33]. Recent reports emphasize a putative association between mitochondrial dysfunction and autism (for review see [34]) and highlight the role of brain energy metabolism dysfunction as an important target for future studies [35]. In ASD, as already emphasized for several neurodegenerative disorders [36], [37], [38], [39], mitochondrial dysfunction could be regarded as a secondary defect in brain energy metabolism.

With the metabolic workup conducted in the sample of 274 patients diagnosed with nonsyndromic ASD, we were unable to detect any of the screened IMD in any of our patients but one, who exhibited persistent urinary excretion of 3-methylglutaconic acid. Our data showed negative results in 99.5% of cases, which did not differ from the estimated prevalence of IMD in the general population [40]. Moreover, the causative relationship between the unspecific 3-methylglutaconic aciduria and ASD is unclear and remains to be proven. These results strongly call into question the utility of a systematic metabolic workup in these patients. Few studies have previously explored the prevalence of IMD associated with ASD. All of them have been performed on restricted cohorts of ASD patients. One study reported that none of the 53 patients with ASD, screened for ammonia, amino acids, lactic acid and pyruvic acid in blood and urinary organic acids, exhibited abnormal results [41]. Also, Wang *et al*. compared the urinary creatine and guanidinoacetate to creatinine ratios between 57 ASD patients and 49 unrelated control samples. They did not detect any significant difference between groups suggesting that creatine deficiency syndromes are not more frequent in patients with ASD than in controls [42].

Several limitations of this study should be underlined. First, we considered as nonsyndromic ASD patients with ID. We assumed that altered mechanisms in the central nervous system in ASD could involve a wide variety of clinical diagnoses crossing categorical boundaries, specifically ID [3]. At the opposite, some authors consider that the co-occurring ASD in association with ID is the consequence of reduced compensatory capacity, and suggested that ASD patients with ID should be regarded as syndromic ASD [43]. Second, the retrospective study design is a limiting factor. Finally, the failure to detect any IMD in our group of patients might be due to the limited sample size; much larger study groups would be warranted to detect extremely rare IMD.

### Conclusions and Perspectives

The data reported here strongly support the view already stressed by others [20], [44] that systematic metabolic investigations are not contributive to the etiology of nonsyndromic ASD. On the other hand, early diagnosis and proper therapeutic intervention for some metabolic disorders causing nonsyndromic ASD may significantly improve the long-term cognitive and behavioral outcomes [20]. Therefore, a careful clinical evaluation, with cautious reappraisal of clinical signs, is crucial. Such a medical practice appears more reasonable than a costly systematic workup. Finally, a large population based prospective study assessing the benefits of routine metabolic screening in nonsyndromic ASD would be of great interest in the future to confirm our results.
