# Correction: Should Metabolic Diseases Be Systematically Screened in Nonsyndromic Autism Spectrum Disorders?

**DOI:** 10.1371/annotation/456e2365-a067-4063-b11b-6a2abeba3f20

**Published:** 2011-08-23

**Authors:** Manuel Schiff, Jean-François Benoist, Sofiane Aïssaoui, Odile Boepsflug-Tanguy, Marie-Christine Mouren, Hélène Ogier de Baulny, Richard Delorme

The 4th author's last name is misspelled. The correct author name is: Odile Boespflug-Tanguy

The correct citation is:

Schiff M, Benoist J-F, Aïssaoui S, Boespflug-Tanguy O, Mouren M-C, et al. (2011) Should Metabolic Diseases Be Systematically Screened in Nonsyndromic Autism Spectrum Disorders? PLoS ONE 6(7): e21932. doi:10.1371/journal.pone.0021932 

